# Reading List: Select Healthcare Transformation Library 2.0

**DOI:** 10.1089/tmj.2020.0399

**Published:** 2021-09-06

**Authors:** Ronald S. Weinstein, Michael J. Holcomb

**Affiliations:** Arizona Telemedicine Program, The University of Arizona, Tucson, Arizona, USA.

**Keywords:** artificial intelligence, digital medicine, e-health, connected health, health care transformation, innovation accelerator, robotics, telehealth, telemedicine

## Abstract

Reading List: Select Healthcare Transformation Library 2.0 represents a broad-based, annotated, general reading list for students of health care innovation. The books were drawn from the 5,000-book private home library of Ronald S. Weinstein, MD, President Emeritus of the American Telemedicine Association. Weinstein is a lifelong book collector with special interests in the history of medical innovation and poetry. A Massachusetts General Hospital-trained pathologist and inductee into the US Distance Learning Association's Hall of Fame, he is known as a pioneer in telemedicine and the “father of telepathology” for his invention, patenting, and commercialization of telepathology, a subspecialty of telemedicine that is a billion-dollar worldwide industry today. This Reading List: Select Healthcare Transformation Library 2.0 consists of 41 books divided into 10 sections: (1) Human Intelligence, Behavior, and Creativity; (2) Societal Revolutions; (3) Innovation; (4) Healthcare System Transformations; (5) Education; (6) Transformational Technologies—Part 1 (AI, Automation, and Robotics); (7) Transformational Technologies—Part 2 (Telemedicine and Telehealth); (8) Digital Medicine; (9) Healthcare Transformation Implementation; and (10) COVID-19 Pandemic as an Innovation Accelerator.

## Introduction

The Select Healthcare Transformation Library 2.0 reading list was created to provide students, clinical investigators, and medical and nurse practitioners with a broad overview of the intellectual landscape in which health care transformation currently takes place and is well suited to serve as a course reading list for students of innovation, health care delivery systems, public health, and medical economics. The books included herein are all part of Dr. Weinstein's extensive personal home library, a collection of over 5,000 books.

Dr. Weinstein's Select Healthcare Transformation Library 2.0 is divided into 10 sections: (1) Human Intelligence, Behavior, and Creativity; (2) Societal Revolutions; (3) Innovation; (4) Healthcare System Transformations; (5) Education; (6) Transformational Technologies—Part 1 (AI, Automation, and Robotics); (7) Transformational Technologies—Part 2 (Telemedicine and Telehealth); (8) Digital Medicine; (9) Healthcare Transformation Implementation; and (10) COVID-19 Pandemic as an Innovation Accelerator. These sections are starting points, not boundaries. Students of health care innovation are well advised to start their journey into the health care transformation literature by giving thought to (1) the topics of human creativity and ingenuity; (2) solutions for current challenges present throughout the health education continuum in the United States; and (3) uses of innovation accelerators to promote societal changes that favor the expansion of digital medicine in the 21st century.

In each of the following 10 sections of books, we have listed 2–8 books that provide information that serves as a springboard for a deep dive into the core of the health care transformation literature and its related topics.

## Human Intelligence, Behavior, and Creativity

- Csikszentmihalyi M. *Creativity: Flow and the Psychology of Discovery and Invention*. HarperCollins Publishers, Inc., 1996, 456 pages.
- Kahneman D. *Thinking, Fast and Slow*. Farrar, Straus and Giroux, New York, NY, 2011, 499 pages.
- Sapolsky RM. *Behave: The Biology of Humans at Our Best and Worst.* Penguin Press, New York, NY, 2017, 790 pages.
- Brandt A, Eagleman D. *The Runaway Species: How Human Creativity Remakes the World*. Catapult, New York, NY, 2017, 296 pages.

Human behavior, intelligence, and creativity help set the stage for the world's great societal transformations. Successful societal health care system transformations will benefit diverse human populations. For an exploration of the creation process, the book by Mihaly Csikszentmihalyi, “*Creativity: Flow and the Psychology of Discovery and Invention*,” is perhaps somewhat dated (as some books often are in any high-quality library), but reading it is also a reminder that earlier scholarship often matters, especially when we think about creativity and the execution of transformative human activities. A masterpiece on human rationality and irrationality by Nobel laureate Daniel Kahneman, “*Thinking, Fast and Slow*” is a must-read for students of the human mind, written by one of the most influential psychologists in the world today. Robert M. Sapolsky's “*Behave: The Biology of Humans at Our Best and Worst*” is a landmark publication that probed the range of human behavior, from the good to the bad. Anthony Brandt and David Eagleman's popular book, “*The Runaway Species: How Human Creativity Remakes the World*,” ponders aspects of human creativity and genius from a historical perspective.

## Societal Revolutions

- McNeill WH. *Plagues and Peoples*. Anchor Press, Garden City, New York, 1976, 329 pages.
- Kuhn TS. *The Structure of Scientific Revolutions*. 3rd edition, University of Chicago Press, Chicago, IL, 1996, 212 pages.
- Schwab K. *The Fourth Industrial Revolution*. Crown Business, New York, NY, 2017.

William McNeill's groundbreaking history book, “*Plagues and Peoples*,” describes how plagues may carry along health care transformations in their wake. McNeill, a distinguished University of Chicago historian, integrated ecology and demography with politics, culture, and the medical science of the day on a grand scale. At the time “*Plagues and Peoples*” was published, in 1976, it was regarded as a fresh look at history, from a unique perspective, by a leading historian.

Thomas S. Kuhn (1922–1996) was a professor emeritus of linguistics and philosophy at the Massachusetts Institute of Technology (MIT) when he published the third edition of his classic publication, “*The Structure of Scientific Revolutions*,” in 1996. This was just before his death and three decades after the publication of its first edition. Kuhn is famous for his recognition that the discovery of anomalies during scientific revolutions leads to new paradigms that ask new questions of old data. Kuhn's classic book had an influence far beyond its original intended audience. It provides one of the best explanations of the processes of discovery.

Celebrations of Klaus Schwab's “*Fourth Industrial Revolution*” are currently the rage among health care leaders who see the confluence of digital technologies, such as artificial intelligence (AI), automation, robotics, and telemedicine, as an innovation-rich river filled with opportunities that humans can harness to solve health care's biggest challenges and to advance us ever closer to the aspirational goal of providing world-class health care for everyone.

## Innovation

- Rogers EM. *Diffusion of Innovations.* 5th edition. Free Press, New York, NY, 2003.
- Moore GA. *Crossing the Chasm. Marketing and Selling Disruptive Products to Mainstream Customers*. HarperCollins Publishers, New York, NY, 2014, 288 pages.
- Christensen CM, Anthony SD, Roth EA. *Seeing What's Next: Using the Theories of Innovation to Predict Industry Change*. Harvard Business School Press, Boston, MA, 2004, 312 pages.
- Christensen CM, Grossman JH, Hwang J. *The Innovator's Prescription: A Disruptive Solution for Health Care*. McGraw Hill, New York, NY, 2008, 496 pages.
- Dyer J, Gregersen H, Christensen C. *The Innovator's DNA: Mastering the Five Skills of Disruptive Innovators.* Harvard Business Review Press, Cambridge, MA, 2011, 272 pages.
- Christensen CM, Grossman JH, Hwang J. *The Innovator's Dilemma: When New Technologies Cause Great Firms to Fail.* Harvard Business Review Press, Cambridge, MA, 2013, 256 pages.
- Ridley M. *The Evolution of Everything: How New Ideas Emerge.* HarperCollins, New York, NY, 2015, 360 pages.
- Poole S. *Rethink: The Surprising History of New Ideas.* Scribner, New York, NY, 2016, 342 pages.

Everett M. Roger's “*Diffusion of Innovations*” is in its fifth edition. It describes what are now regarded as the five classic stages in the development and diffusion of innovations. Roger was an eminent American communication theorist and sociologist who originated the diffusion of innovations theory. He explained how innovations spread through populations in stages, along the line of a five-stage bell-shaped curve. His stages are (1) “Innovators”; (2) “Early Adopters”; (3) “Early Majority”; (4) “Late Majority”; and (5) “Laggards.” Geoffrey A. Moore's “*Crossing the Chasm. Marketing and Selling Disruptive Products to Mainstream Customers*” focuses on overcoming the chasm that may be encountered in the rollout of Christensen's disruptive innovations between the “Early Adopters” and “Early Majority” stages in Roger's bell-shaped curve in the technology adoption life cycle.

Clayton M. Christensen, a Harvard Business School distinguished professor, was a thought leader in innovation science for many years. Five of Christensen's books are included in this 41-book Select Healthcare Transformation Library. His “*The Innovator's Prescription: A Disruptive Solution for Health Care*” describes a wellspring of ideas that add to our understanding of how successful products and services may evolve. In essence, it provides a roadmap for reinventing health care delivery systems in the United States. Christensen's “*The Innovators DNA: Mastering the Five Skills of Disruptive Innovators*” coaches innovators on what it takes to succeed in making their enterprises work. His “*The Innovator's Dilemma: When New Technologies Cause Great Firms to Fail*” describes strategies for maintaining a competitive edge at companies. The fifth Christensen book selection is listed in “Section 5. Education.”

Matt Ridley's “*The Evolution of Everything: How New Ideas Emerge*” and Steven Poole's “*Rethink: The Surprising History of New Ideas*” are interesting reads that highlight the broad range of sources of new ideas, including the recycling of older ideas, which pop up as newer innovations within a later time frame.

## Healthcare System Transformations

- Blumenthal D, Morone JA. *The Heart of Power: Health and Politics in the Oval Office*. University of California Press, Berkeley, CA, 2009, 484 pages.
- Emanuel EJ. *Reinventing American Health Care: How the Affordable Care Act Will Improve our Terribly Complex, Blatantly Unjust, Outrageously Expensive, Grossly Inefficient, Error Prone System.* Public Affairs, New York, NY, 2014, 380 pages.
- Haseltine WA. *World Class: A Story of Adversity, Transformation, and Success at NYU Langone Health.* Fast Company Press, New York, NY, 2019, 340 pages.

David Blumenthal and James A. Morone's “*The Heart of Power: Health and Politics in the Oval Office*” is a presidential history book that takes the reader through the evolution of health care policy during 11 US presidencies, starting with Franklin D. Roosevelt and continuing through George W. Bush's presidency. It shows the centricity of the US president in the decision-making that affects the health care of large swaths of the US population.

Ezekiel J. Emanuel's “*Reinventing American Health Care: How the Affordable Care Act Will Improve our Terribly Complex, Blatantly Unjust, Outrageously Expensive, Grossly Inefficient, Error Prone System*” is recommended for readers interested in understanding how the US health care system functions and wishing to tackle transforming our byzantine US health care system into something new that functions well and fairly for all Americans. It is a detailed case study of how a brilliantly conceived health care policy, and its enabling legislation, can accomplish health care transformation even within a highly fragmented health care delivery system. It gave reason for hope for the continuing of US health care system transformation processes in positive directions. On the other hand, it turns out that this hope may have been premature.

William A. Hazeltine's book, entitled “*World Class: A Story of Adversity, Transformation, and Success at NYU Langone Health*,” is an inspiring account of the recent bailout and so-called transformation of a large university health care system in New York City. The rehabilitation process at New York University Langone was led by a visionary, arguably a one-of-a kind, New York City billionaire, who achieved total personal immersion in his mission to make New York University Langone the best-of-breed health care system in the New York City area. He succeeded according to a number of measures of excellence. This remarkable, academic, health care system rehabilitation story deserves special mention in the hopes that it will inspire other philanthropists to make comparable investments in our future. As this was happening, New York City emerged as a significant player in the development of leading-edge telemedicine-enabled reorganizations of vast, regional, health care delivery systems. That spirit of change spread to nearby Philadelphia, especially at Jefferson University, in even more recent times.

## Education

- Christensen CM, Horn MB, Johnson CW. *Disrupting Class: How Disruptive Innovation Will Change the Way the World Learns*. McGraw Hill, New York, NY, 2008, 238 pages.
- Bowen WG, Chingos MM, McPherson MS. *Crossing the Finish Line: Completing College at America's Public Universities*. Princeton University Press, Princeton, NJ, 2009, 389 pages.
- Fisher JF, Fisher D. *Who You Know: Unlocking Innovations that Expand Students' Networks*. Jossey-Bass, San Francisco, CA, 2018, 180 pages.

Clayton M. Christensen, Michael B. Horn, and Curtis W. Johnson, in “*Disruptive Class: How Disruptive Innovation Will Change the Way the World Learns*,” take Christensen's earlier ideas on business disruptive innovation and expand on them in the context of education reform. Christensen, a brilliant teacher at Harvard Business School, along with a pair of coauthors, takes a stab at identifying ways to improve education research. Giving Schools the Right Structure to Innovate, the last chapter in the book, needs updating, especially in light of the explosive growth of distance learning in response to the COVID-19 pandemic early in 2020. Currently, the world seems overwhelmed with mandates to teach K-12 students at home in front of computer screens and make that equivalent to the traditional in-classroom experience. It is a time when we need our great thinkers hard at work to create education schemas that employ novel methods and leverage communication technologies to overcome physical location and bring students together in ways that improve their educational experience and outcomes. Sadly, Harvard Business School Professor Clayton Christensen died on January 23, 2020, at age 67. He will be missed as one of original thinkers in the business innovation arena, and beyond.

William G. Bowen, Matthew M. Chingos, and Michael S. McPherson's “*Crossing the Finish Line: Completing College at America's Public Universities*” is a sobering discussion of the challenges facing state universities in the United States. The ambivalence of state legislatures toward funding their state universities is a significant societal challenge.

Finally, the book, “*Who You Know: Unlocking Innovations that Expand Students' Networks*,” authored by Julia F. Fisher with Daniel Fisher, highlights the importance of engagement and networking in many human activities. The 16-page introduction to the book was authored by Clayton Christensen. The critical student needs for learning, referred to as soft skills, including networking skills, are becoming targets of interest and concern among educators. Are networking skills sacrificed in distance learning activities? Should explicit training in soft skills be added to the curriculums of health profession schools? Is it important to learn how to network with influential people? At what grade levels in the education curriculum should attention be directed at teaching of networking skills? Are teachers in the current workforce prepared and up to the task?

## Transformational Technologies—Part 1 (AI, Automation, and Robotics)

- Evans H. *They Made America: From the Steam Engine to the Search Engine: Two Centuries of Innovators.* Little, Brown and Company, New York, NY, 2004, 496 pages.
- Ford M. *Rise of the Robots: Technology and the Threat of a Jobless Future*. Basic Books, New York, NY, 2015, 334 pages.
- Mindell DA. *Our Robots, Ourselves: Robotics and the Myths of Autonomy*. Viking, New York, NY, 2015, 360 pages.
- Topol E. *Deep Medicine: How Artificial Intelligence Can Make Healthcare Human Again.* Basic Books, New York, NY, 2019, 378 pages.
- Russell S. *Human Compatible: Artificial Intelligence and the Problem of Control*. Viking Press, New York, NY, 2019, 336 pages.

Harold Evans's highly readable anthology shows how emerging technologies can drive the creation of new industrial revolutions and their social transformations. Currently, we are witnessing astonishing advances in computer science, robotics, AI, and automation as major drivers of transformation in society across many business sectors. Martin Ford's “*Rise of Robots: Technology and the Threat of a Jobless Future*” provides a vision of what impact robots could have on the job market. David A. Mindell's “*Our Robots, Ourselves: Robotics and the Myths of Autonomy*” questions the impact of robots on society in the future. Eric Topol's “*Deep Medicine*” pictures health care systems becoming highly influenced by the intrusion of AI and deep learning, even overarching influences on health care delivery sometime in the future. Stuart Russell's “*Human Compatible: Artificial intelligence and the Problem of Control*” explains the risks to humanity of increasingly powerful AI programs over time. Is there a point at which AI becomes the master of us all?

“*Deep Medicine*” provides an enlightened description of Topol's personal, virtual multiyear tour of the AI and deep learning industries. He has a keen eye for detail and succeeds in weaving together a coherent picture of a future health care industry emerging from the integration of hundreds of AI-enabled apps that are under construction today. The final chapter, entitled “Deep Empathy,” seems heartfelt and highly personal. It is inspiring to have a practicing doctor this engaged in computer-based medicine while, apparently, retaining loyalty to his roots as a traditional hands-on physician. Dr. Topol remembers where he came from.

The final chapter in “*Deep Medicine*” describes how medical care could positively develop in a technology-enabled world, as seen through the eyes of an optimist. Topol describes his transformative vision for the medical practice environment of the future, one in which doctors are freed up from their back-office menial tasks (scheduling appointments, billing for services, and electronic health record chart reviews, etc.) and personally return to pure caring for patients. Hopefully, medical science and medical humanities would undergo what sounds a lot like what is called convergent evolution. There, different species begin to look alike from their cohabitation in a shared environment.

Dr. Topol is eminently qualified as a candidate for the title of “Modern-day Sir William Osler,” as both an empathic physician and an accomplished medical scientist. We recommend that medical students read Dr. Topol's last chapter in “*Deep Medicine*” (Chapter 13), entitled “Deep Empathy,” first. We would like medical students to have empathy foremost in their minds as they do a deep dive into AI. In Dr. Topol's last chapter, he describes what medical practice could look like when the thousands of computer-enabled individual components of the health care delivery system become interconnected into a single, interconnected digital landscape that would free up doctors' time and become an enabler for the practice of truly patient-centric health care once again. Hopefully, some of that happens!

## Transformational Technologies—Part 2 (Telemedicine and Telehealth)

- Crichton M. *Five Patients: The Hospital Explained.* Alfred A. Knopf, New York, NY, 1970, 239 pages.
- Bashshur RL, Shannon GW. *History of Telemedicine: Evolution, Context, and Transformation.* Mary Ann Liebert, Inc., Publishers, New Rochelle, NY, 2009, 415 pages.
- Vladzymyrskyy A, Jordanova M, Lievens F. *A Century of Telemedicine*. Jordanova Publisher, Donetsk, Ukraine, 2016, 341 pages.
- Dumanskyy YV, Vladzymyrskyy A, Lobas VM, Lievens F. *Atlas of the Telemedicine History*. Jordanova Publisher, Donetsk, Ukraine, 2013, 72 pages.
- Wootton R, Craig J, Patterson V. *Introduction to Telemedicine*, 2nd edition, CRC Press, Boca Raton, FL, 2017, 197 pages.
- Rheuban KS, Krupinski EA. *Understanding Telehealth*. McGraw-Hill, New York, NY, 2018, 305 pages.
- Yellowlees P, Shore JH. *Telepsychiatry and Health Technologies: A Guide for Mental Health Professionals*. American Psychiatric Association Publishing, Arlington, VA, 2018, 381 pages.

In 1969, Michael Crichton, MD, the author of Andromeda Strain, and future author of Jurassic Park, was the first Harvard Medical School (HMS) medical student to rotate through the Logan International Airport–Massachusetts General Hospital (MGH) Medical Station telemedicine program and write a book chapter about it. His first nonfiction book “*Five Patients: The Hospital Explained*” is the book in which HMS senior student Crichton tracked five patients he encountered at the MGH, including one telemedicine patient. The book became a popular success and was an alternative book club selection. Crichton's book provides a rare first-person account of a Harvard medical student's initial exposure to several then-futuristic medical innovations, including computer-enabled, automated patient history taking and telemedicine (then called telediagnostics), during his own medical school 4th-year clinical rotations. This was in the late 1960s, a half century ago. Crichton left medicine upon graduation from HMS. He never practiced medicine, but went on to become a famous science fiction writer and movie director. Nevertheless, Crichton retained his interest in progress in medical science throughout his life.

In his “*Five Patients: The Hospital Explained*” book, Crichton recounts his telemedicine patient encounter with a Logan Airport traveler, as a medical student stationed 2.7 miles away in the MGH Telediagnosis Center on the first floor of the White Building on the MGH campus, in remarkable detail. Crichton's telemedicine patient, Mrs. Sylvia Thompson, was a passenger flying from Los Angeles to Boston when she developed chest pain while in the air over Ohio flying east. Upon landing, she walked to the Logan Airport MGH Medical Station walk-in clinic near Gate 23 and was examined in the Teleconsultation Room at the Medical Station by a telephysician located at the MGH 2.7 miles away. At the end of her telemedicine physical examination, Mrs. Thompson exclaimed, “*My goodness. It was just like the real thing.*” Her immortal words, uttered at the Logan Airport walk-in clinic, were likely repeated by millions of new telemedicine patients a half century later during the COVID-19 public health emergency as health care providers rushed to utilize telemedicine as a means to continue providing health care to their patients while leveraging telemedicine's inherent social distancing to mitigate the spread of COVID-19.

Following the rendering of her provisional diagnosis of pneumonia by a telephysician at the MGH in Boston, Mrs. Thompson was transported to the MGH for X-rays and treated for pneumonia following confirmation of her diagnosis by X-ray examination. It is interesting that television microscopy, the forerunner to modern telepathology, was available at the Logan Airport MGH Medical Station from the start of their telemedicine program in April 1968. Teleradiology became available at the MGH Medical Station over a year later. A decade of prior experience by television microscopy research investigators had validated the video microscopy technology. Television microscopy was ready for immediate implementation in clinical practice.

Crichton expands on his medical student telemedicine patient experience with a remarkably insightful consideration of a series of futuristic medical service innovations that would leverage leading-edge technologies, including private telecommunications networks, telemedicine, computers, and even AI. Based upon his acknowledgments in his book “*Five Patients*,” it is likely that Crichton, the soon-to-be best-selling science fiction writer Michael Crichton, had extracted nuggets of sophisticated information from a series of personal interviews with leading, senior faculty members at the MGH and at MIT across the Charles River from the MGH campus. It is likely that some of these high-profile interviews had been arranged by Dr. John H. Knowles, the General Director at the MGH. Crichton, already a legendary summa cum laude graduate from Harvard College, whose reputation as a brilliant undergraduate student preceded him at HMS, had the instincts of an investigative reporter, which he had acquired from his journalist father at a young age. The younger Crichton had published his first article, one on travel, in the New York Times at age 14, and as a physically imposing 26-year-old, Crichton cut an extraordinary figure.

As a footnote to history, Weinstein, the coauthor of this article, and Michael Crichton were contemporaries at the MGH during their training. Although Weinstein was four academic grades ahead of Crichton, they were MGH trainees at overlapping periods of time in the late 1960s (1968–1969). Weinstein vividly remembers seeing the towering, 6-foot 9-inch Michael Crichton strolling through the MGH corridors accompanied by a relatively young, and equally charismatic, 5-foot 8-inch MGH General Director, John H. Knowles, MD. Dr. Knowles was an early proponent for telemedicine. Crichton and Knowles were seen either walking together or having lunch in the MGH employee cafeteria many times. Weinstein recalls seeing them engrossed in serious discussions: an HMS medical student and the youthful hospital director, Crichton and Knowles, a Socratic pairing always acting out in public, face-to-face, despite their striking differences in physical stature. They were a memorable pair to observe in person, even at a distance. High energy appeared to flow naturally between the two of them.

Rashid L. Bashshur and Gary W. Shannon's “*History of Telemedicine*” is the product of meticulous scholarship by a pair of accomplished historians who display a special talent for explaining the complex interrelationships between people, events, and technologies. Bashshur is more than a historian. He has been professionally immersed in telemedicine since the 1960s, arguably longer than almost anyone else alive today. He has chronicled major events in the field in real-time, up close and personal, while authoring a significant part of the analytical literature on many facets of this complex health care delivery field with a “who's who in the field” panel of collaborators. Bashshur and Shannon's particular interest in public policy is reflected in their insightful descriptions of societal influences on the jerky progression of the implementation of telemedicine, particularly in North America, over half a century. The recent surge in COVID-19 pandemic-related, telemedicine case activity becomes understandable in terms of the decades-long deadweight influences of policy on earlier efforts by an army of workers to implement telemedicine and take it to scale.

The first half of the book is a detailed recounting of the adaptations of groundbreaking technical innovations for uses of telemedicine in health care, dating back literally to the Trojan War. Much of the more recent history is told against the backdrop of the birth and expansion of a new nation. The descriptions of innumerable events and activities are often riveting and even majestic. There is great precision to the story telling, which adds to its credibility. Bashshur and Shannon shift gears in the second half of the book by focusing the second half of the book on the modern era of telemedicine in the United States (1950–2009) and, to a greater extent than in the first half of their book, on the influences of public policy on telemedicine adoption and expansion. They carefully select model programs to illustrate specific points, such as roles for government in creating successful telemedicine programs.

A limitation of Bashshur and Shannon's “*History of Telemedicine*” is its under-reporting of telemedicine studies published in foreign language journals. Sponsorship of the book by the US National Library of Medicine may have played a role in limiting the scope of the book. It is noteworthy that there is a wealth of telemedicine and telehealth information in many foreign language journals. This is published in abundance in publications of the International Society of Telemedicine and Telehealth. Their publication reference citations and titles are dual published in the foreign language of origin and also in English translations.

For a greater international perspective on the development of telemedicine around the world, check out “*A Century of Telemedicine: Curatio Sine Distantia et Tempora*,” edited by Anton Vladzymyrskyy, Malina Jordanova, and Frank Lievens and published in 2016. This book is more of a survey than a history, and it is quite narrowly focused on technologies. The book's six chapters' titles include telecommunications, videoconferencing, telecardiology, biotelemetry, computational telediagnosis, and satellite technologies. Although titled in two languages, the detailed text is written entirely in English.

“*Century of Telemedicine*” is profusely illustrated. It is fun to see page after page of thumb-nail sized pictures illuminating many surprises, not the least of which is how many other countries have had telemedicine-related developments for decades. Unlike Bashshur and Shannon's “*History of Telemedicine*,” there is barely any description of national health care systems, societal priorities, or telemedicine applications that have gone mainstream. We now know that was by design since “*Century of Telemedicine*” has been followed up with a three-volume set of books specifically on over a dozen individual national telemedicine programs (see below). This tends to debunk the popular US notion that “telemedicine started here!”

“*Century of Telemedicine*” also includes an extensive interesting Appendix (called an Afterword) listing short bibliographies for historic participants in telemedicine programs. Included are descriptions and photographs for a large sampling of participants in telemedicine from the United States and Canada. Coverage is uneven and the criteria for inclusion are not stated. There are many obvious omissions.

“*Century of Telemedicine*” is a product of the International Society for Telemedicine and Telehealth (ISfT). ISfT was officially founded in 1997 after several failed starts at several international meetings. The ISfT was officially registered in Basel, Switzerland, in 1997, with its Coordinating Office presently in Belgium.

Dr. Weinstein, coauthor of this article, delivered the opening lecture at the “First International Conference on the Medical Aspects of Telemedicine,” held in Tromsø, Norway, in May 1993. This is where the seed was planted for ISfT. Dr. Weinstein gave the opening keynote address at the Tromsø Conference, on the topic, “Telemedicine in the United States.” The Conference Chair acknowledged Dr. Weinstein's invention of robotic telepathology in 1986 and its successful implementation in northern Norway, above the Arctic Circle, 3 years later. Because of this, Dr. Weinstein was well known in the European pathology community. His invention had been successfully implemented by Tromsø University Hospital pathologists as an enabling telemedicine technology that supported intraoperative frozen section diagnosis at Kirkenes Hospital, 400 km to the east. This was over a two-way E1 2 megabit per second landline, the European version of a US carrier T1 1.5 megabit per second landline.

ISfT was renamed the International Society for Telemedicine and eHealth (ISfTeH) in 2005. Today, there are 90 countries participating in ISfTeH. The organization has robust education and publication programs. Its publications are authoritative and of generally high quality.

In retrospect, the original “*A Century of Telemedicine*” book now serves as an introductory book for a set of ISfTeH-endorsed books covering telemedicine programs in individual countries. The three books in this new series provide detailed descriptions of telemedicine programs in 16 countries: Book 1 (Australia, Brazil, Czech Republic, India, Nigeria, and Russia); Book 2 (Chile, Finland, Georgia, Japan, Peru, and United States); and Book 3 (Bolivia, Denmark, Iran, and Poland). The chapter on the United States in Book 2 is telenurse oriented and may disappoint physicians. Generally, comparisons of the telemedicine programs in individual countries, by the reader, might show how different national and cultural points of view influence countries' development and implementation of telemedicine and telehealth services. Each country has its own attitudes toward the well-being of its citizens. This can be reflected in the structures and functions of their telemedicine programs. However, such comparisons are somewhat complicated by the diversity of professions of the authors of the chapters (medical doctors and nurses, etc.). It would be helpful of ISfTeH to add a fourth book to the series, authored by a panel of experts assigned the task of analyzing the chapters describing telemedicine and telehealth in 16 individual countries and of identifying and discussing common threads. This ISfTeH global telemedicine book series calls out for analyses, and discussion, by authorities with a broad international perspective.

A fifth ISfTeH-endorsed telemedicine book is a special gem. This 72-page “*Atlas of the Telemedicine History*,” published in 2013, contains many rare historical photos of early telemedicine pioneers and their programs from, literally, around the world. This is an outstanding, historical picture gallery.

PDFs for these ISfTeH-promoted books are available, for free, at the ISfTeH webpage.^1^

Currently, there is a crescendo of interest in potential telemedicine and telehealth textbooks, worldwide. In the United States, schools, ranging from community and undergraduate colleges to a wide range of graduate schools, will be offering telehealth courses this year for the first time. The COVID-19 pandemic appears to be functioning as an innovation accelerator in the distance education industry.

Due to the COVID-19 pandemic, in the United States, telemedicine caseloads in medical practices have increased at astronomical rates, not infrequently exceeding 3,000–6,000% for many medical practices in the United States. The vast majority of US practitioners had no telemedicine in their medical school training. Many office practices went from zero telemedicine cases to 1,000s of telemedicine cases per month in a matter of a month or two.

The Association of American Medical Colleges, which oversees medical student education in the United States, has announced an urgent need to generate core competencies for medical students in virtual care, telemedicine, and telehealth. Other categories of health care profession schools have overlapping needs. A race is on to identify telehealth textbooks best suited to address trainees' needs. Potential contenders, already on the market, are worth mentioning.

Richard Wootton, John Craig, and Victor Patterson's second edition of their “*Introduction to Telemedicine*” was published in 2017. The book's 17 contributors are drawn from five different countries on three continents. This reflects R. Wootton's extraordinary reach as an international authority on telemedicine. The second edition of Wootton's “*Introduction to Telemedicine*” lends itself to being a quick read for medical students or even practicing physicians who are exploring telemedicine for the first time. One drawback to using Wootton's “*Introduction to Telemedicine*” is that many of the pictures and figures are holdovers from the first edition and need updating.

Wootton's “*Introduction to Telemedicine*,” published by RMS Press, was designed to serve as a general introduction for what became a seven-book series of specialty telemedicine books. His “*Introduction to Telemedicine*” can be used either as a stand-alone textbook for an introductory telemedicine course or in combination with Wootton's advanced topic textbooks for multisemester courses. The introductory medical specialty books in Wootton's seven-book collection cover specific telemedicine applications, including telepsychiatry, telepediatrics, teleneurology, and teledermatology, quite well, but need updating.

The publication of “*Understanding Telehealth*,” edited by Karen S. Rheuban and Elizabeth A. Krupinski, was a major event for the telemedicine and telehealth industry. For the sake of disclosure, Dr. Krupinski and Dr. Weinstein have been colleagues and close collaborators since 1992.

That being said, Rheuban and Krupinski's book is brilliantly conceived and masterfully executed by any measure. “*Understanding Telehealth*” consists of 24 chapters, authored by 46 experts in the telemedicine field. Telehealth, per se, which is inclusive of telemedicine, is well represented. Chapter titles range from “Telehealth in Pediatric Cardiology” to “Legal and Regulatory Issues.” Despite their breadth of subject matter coverage, individual chapters are remarkably uniform with respect to the breadth of coverage of individual topics and their readability.

In addition to providing essential foundational information for a broad swath of health care workers new to telemedicine, Rheuban and Krupinski's “*Understanding Telehealth*” seamlessly kernels in what amounts to select up-to-date microreviews on a broad range of current topics of interest.

“*Understanding Telehealth*” is highly recommended for a broad spectrum of learners and professionals, including nonmedical staff, medical, nursing, and pharmacy students, residents, medical practitioners, public health officials, and C-suite health care executives, seeking updates on the issues of the day. “*Understanding Telehealth*” might not be a suitable textbook for a college Gen Ed course or an introductory course in an allied health profession curriculum.

Currently, “*Understanding Telehealth*” is the telemedicine reference book of choice for our staff members at the Arizona Telemedicine Program. In addition, in our experience, some sophisticated patients and their advocates might find sections of the book worth reading. Finally, one can imagine that healthcare portfolio managers at financial institutions would be interested in diving into “*Understanding Telehealth*” with their eyes wide open. There is a great deal of information they will find worth mining in light of so many investment opportunities in telemedicine and telehealth these days! Outstanding nonfiction books such as this can generate large readerships.

My ideal telemedicine and telehealth textbook for undergraduate college students remains to be written. Frankly, if I had access to a suggestion box, this textbook would be a generalized version of Peter Yellowlees and Jay H Shore's “*Telepsychiatry and Health Technologies: A Guide for Mental Health Professionals.*” Book collectors do get to fantasize and, occasionally, dreams do come true. Although marketed as a telepsychiatry book, which it is, much of the information in “*Telepsychiatry and Health Technologies: A Guide for Mental Health Professionals*” is not psychiatry specific and could be of value to students planning careers in nearly every sector of the health care industry. In general, according to our analysis of the book, nonpsychiatry information in the book could be carved out and repackaged as a general textbook on telemedicine and telehealth that could be outstanding. That might be of great immediate use to faculties at US medical schools, and undergraduate colleges, which are in urgent need of a high quality, US-centric, general telemedicine textbook because of the COVID-19 pandemic. We can see how a revised, generalized, and shortened version of “*Telepsychiatry and Health Technologies: A Guide for Mental Health Professionals*” could fit that bill.

It is noteworthy that Yellowlees and Shore's strategy of developing their model textbook is clearly stated on the first page of their preface. This could be replicated by others willing to take the time to do so. Instead of giving recruited authors free reign in selecting their designated chapter's subject matter and then simply assembling their chapters, as is often the case, Yellowlees and Shore “initially mapped out the book down to the section headings…and then selected colleagues to write the various sections of the book based on their individual expertise and knowledge.” There it is! That is the way to do it. Their product is an excellent textbook for a course on telemedicine, created by master educators and executed in partnership with a team of carefully selected high-quality scholar educators willing to follow their coeditor's directions. For the medical reader, telepsychiatry becomes a metaphor for a broad spectrum of medical specialties.

## Digital Medicine

- Topol E. *The Creative Destruction of Medicine: How the Digital Revolution Will Create Better Health Care.* Basic Books, New York, NY, 2011, 320 pages.
- Topol E. *The Patient Will See You Now: The Future of Medicine is in Your Hands*. Basic Books, New York, NY, 2015, 374 pages.
- Wachter R. *The Digital Doctor: Hope, Hype, and Harm at the Dawn of Medicine's Computer Age*. McGraw Hill, New York, NY, 2015, 330 pages.
- André A. *Digital Medicine*. Springer International Publishing, New York, NY, 2018, 115 pages.

What is the future digital health care ecosystem once the current wave of innovations in digital technologies is in full blossom? What might the health care system look like? Where are we if, and when, the traditional doctor–patient relationship, which has provided the backbone for health care systems (as we knew them for past centuries), is a thing of the past? Robert Wachter's “*The Digital Doctor*” discusses the possibilities for progress in a digital age from the perspective of a medical practice leader deeply embedded as a practicing physician in San Francisco. “*Digital Medicine*,” edited by Arthur André, gives a French-flavored update on digital medicine. Chapters focus on AI, big data applications, blockchain, regenerative medicine, and telemedicine. Dr. André is an enlightened neurosurgeon practicing in Paris, France.

## Healthcare Transformation Implementation

- Brownson RC, Colditz GA, Proctor EK. *Dissemination and Implementation Research in Health: Translating Science to Practice.* Oxford University Press, New York, NY, 2018, 515 pages.
- Chambers DA, Vinson CA, Norton WE, eds. *Advancing the Science of Implementation across the Cancer Continuum.* Oxford University Press, New York, NY, 2019, 409 pages.

Ross C. Brownson, Graham A. Colditz, and Enola K. Proctor's “*Dissemination and Implementation Research in Health*” and David A. Chambers, Cynthia A. Vinson, and Wynne E. Norton's “*Advancing the Science of Implementation across the Cancer Continuum*” are two recently published books on dissemination and implementation research, both published by Oxford University Press. Brownson et al.'s book was the first of its kind: a practical textbook on dissemination and implementation research. Both books rapidly became required reading in the clinical dissemination and implementation of research in the health care field. These books are essential reading for clinical investigators and students interested in carrying out US federally funded intervention studies. They are also suitable for use as classroom textbooks on “how to do it.”

The book, “*Dissemination and Implementation Research in Health: Translating Science to Practice*,” is inclusive and covers a broad spectrum of topics ranging from introductory terminology and the historical roots of dissemination and implementation research to dissemination and implementation research in a global context. There are 29 multiauthor chapters. Discussions of complex issues are presented in easy-to-understand language. This is noteworthy in light of the interdisciplinary nature of their target audiences.

Chambers et al.'s “*Advancing the Science of Implementation across the Cancer Continuum*” can be viewed as an extension of Brownson et al.'s “*Dissemination and Implementation Research in Health*.” In addition to reiterating, and expanding on, introductory material in Brownson et al.'s “*Dissemination and Implementation Research in Health*” and expanding on certain topics, such as the history of NIH funding for dissemination and implementation research (see page 18), Chambers et al.'s book focuses on a single disease entity, cancer, and includes a series of chapters describing 19 highly informative case studies. These case studies will be of general interest.

We hope that publication of this important pair of Oxford University Press books, by top authorities in the dissemination and implementation research fields, will increase the visibility of dissemination and implementation research and help catalyze a further expansion of funding for dissemination and implementation research by governmental agencies and foundations. Leaders in academic medicine would do well to read these books themselves and jump on board as advocates for this often undervalued component of health care transformation processes.

## COVID-19 Pandemic as an Innovation Accelerator

- Barry JM. *The Great Influenza: The Story of the Deadliest Pandemic in History*. Penguin Books, New York, NY, 2004, 546 pages.
- Osterholm MT, Olshaker M. *Deadliest Enemy: Our War Against Killer Germs*. Little, Brown Spark, New York, NY, 2020, 314 pages.

The COVID-19 pandemic could turn out to be an innovation accelerator of historic proportions. Expect a flood of books on innovations resulting from the COVID-19 pandemic in the foreseeable future.

To help prepare for reading the COVID-19 pandemic-related literature, two outstanding books are John M. Barry's “*The Great Influenza: The Story of the Deadliest Pandemic in History*,” published in 2004, and Michael T. Osterholm and Mark Olshaker's “*Deadliest Enemy: Our War Against Killer Germs*,” published in 2017, with an updated foreword in 2020.

Barry recounts the development of scientific theories and their importance in creating new diagnostic technologies and cures in the early 20th century. Newer scientific theories became foundational for the evolution of modern medicine. In “*Deadliest Enemy: Our War Against Killer Germs*,” Osterholm and Olshaker describe how nations can prepare for a pandemic and avert disastrous population outcomes by sticking to scientific-based reasoning. Their message is that health care catastrophes are preventable, but scientific discovery is an essential component of the process.

## About the Authors

Ronald S. Weinstein, MD, FCAP, FATA, is an MGH-trained pathologist. He is the Founding Director of the Arizona Telemedicine Program and a President Emeritus of the American Telemedicine Association. He invented, patented, and commercialized telepathology and introduced the term “telepathology” into the English language.

Mr. Michael Holcomb, BS, is the Associate Director for Information Technology in the statewide Arizona Telemedicine Program. He lectures on telemedicine technology and information security. Mr. Holcomb and Dr. Weinstein have coauthored dozens of articles together.
